# Electrospray deposition of organic molecules on bulk insulator surfaces

**DOI:** 10.3762/bjnano.6.195

**Published:** 2015-09-18

**Authors:** Antoine Hinaut, Rémy Pawlak, Ernst Meyer, Thilo Glatzel

**Affiliations:** 1 Department of Physics, University of Basel, Klingelbergstrasse 82, 4056 Basel, Switzerland

**Keywords:** adsorption, electrospray, insulating surface, large molecules, non-contact AFM, ultra-high vacuum (UHV)

## Abstract

Large organic molecules are of important interest for organic-based devices such as hybrid photovoltaics or molecular electronics. Knowing their adsorption geometries and electronic structures allows to design and predict macroscopic device properties. Fundamental investigations in ultra-high vacuum (UHV) are thus mandatory to analyze and engineer processes in this prospects. With increasing size, complexity or chemical reactivity, depositing molecules by thermal evaporation becomes challenging. A recent way to deposit molecules in clean conditions is Electrospray Ionization (ESI). ESI keeps the possibility to work with large molecules, to introduce them in vacuum, and to deposit them on a large variety of surfaces. Here, ESI has been successfully applied to deposit triply fused porphyrin molecules on an insulating KBr(001) surface in UHV environment. Different deposition coverages have been obtained and characterization of the surface by in-situ atomic force microscopy working in the non-contact mode shows details of the molecular structures adsorbed on the surface. We show that UHV-ESI, can be performed on insulating surfaces in the sub-monolayer regime and to single molecules which opens the possibility to study a variety of complex molecules.

## Introduction

Large complex molecules with tunable electronic properties are building block candidates for functional materials with special electrochemical and photophysical properties, which are of fundamental interest for many applications such as hybrid-photovoltaic [1] or molecular electronics [2]. Information at the single molecular level, even if challenging, is required to foresee the interplay between nanoscale structures and geometries and the device properties. For reliable investigations of such systems, a well defined environment is necessary and therefore ultra-high vacuum (UHV) conditions are required for these fundamental studies. However, thermal evaporation, the most commonly employed technique under UHV conditions, may lead to a fragmentation of large molecules generally happening before reaching the sublimation temperature. Therefore the study of such complex molecules with high resolution and precision is hindered. Recently, other deposition techniques have been introduced, e.g., direct deposition from a liquid solution (droplet casting) on a freshly prepared surface or pulsed valve deposition in UHV. Although these techniques are compatible with molecular resolution, the pollution from solvents remains a problem [3–5].

Electrospray ionization (ESI), first developed by Fenn et al. [6] allows for the introduction of large organic molecules in vacuum. Originally developed for mass spectrometry and protein studies, it has since been used with many other types of molecules. In ESI, molecules are directly ionized from solution, allowing to select and analyze them with electrostatic lenses. Following this initial use, numerous experimental setups have been built to deposit such ionized molecules on surfaces. For such ESI deposition systems, the combination with filtering devices, made of quadrupoles, octopoles and other electrostatic stages allows one to filter ions and guide them towards the sample surface [7–10]. An advantage of theses systems, in addition to the selection of the ion species, is the use of a soft landing deposition where additional electrostatic lenses are used to reduce the kinetic energy of the molecules [11–13]. Moreover it lowers the chance of fragmentation by impacting surfaces. However the complexity of the setup as well as the proper adjustment somehow limits their usability.

An easy and commercially available ESI system has already proven its capability to deposit simple [14–15] or more complex molecules [16–20] on various surfaces such as metals or TiO_2_. The setup is aligned in straight line and no selection or deviation elements are used. As a result, all species introduced in vacuum that are not pumped are directed towards the surface. Consequently, the use of contaminant-free solvent, as well as appropriate spray parameters is primordial to successful deposition of single molecules.

With the prospect of studying the intrinsic properties of large molecules, their decoupling from the metal surface is desirable. Deposition and analysis on insulating films or crystals is thus mandatory and requires high resolution imaging. Therefore, for the characterization at the atomic-scale, atomic force microscopy (AFM) is mandatory. Numerous experimental AFM studies have shown the possibility to image molecular islands [21–27], small aggregates [28], single molecules [29] as well as trapped single molecules [30–32] at room temperature on insulating surfaces. The use of ESI now allows one to study even larger and more complex molecules which are more suitable for future devices and could incorporate additional functions and anchoring groups.

In this work we present the adaptation of a UHV-ESI system to deposit triply fused porphyrin molecules on a bulk insulator KBr(001) sample and the analysis of theses deposits by high resolution AFM measurements at RT and under UHV conditions. First, it is shown that the applied solvents do not impact the deposits and the measurements. The coverage of the molecules on the bulk insulator surface can be controlled and adopted to the needs of the AFM measurement. However, at large coverage, charging of the surface was observed, which could be successfully overcome by a moderate annealing of the surface. The formation of various molecular assemblies was achieved and even isolated molecules could be analyzed at room temperature.

## Results and Discussion

### UHV-ESI of solvent

**Figure 1:**
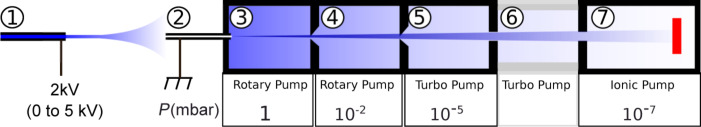
Scheme of the commercial ESI setup [33] (1 to 5) connected to the UHV chamber (7), i.e., sample preparation chamber. The additional vacuum chamber (6) was added to further enhance the performance of the deposition system.

UHV-ESI has been performed with a modified commercial MolecularSpray setup [33], of which a scheme is shown in Figure 1. The mixture of solvent and molecules is introduced by a syringe pump and a needle (1) into the first vacuum chamber (3) trough a capillary (2) by applying a bias of several kilovolts (1 to 5 kV). Behind the entrance capillary, three chambers (3, 4, 5) are used to pump solvent molecules and to reach the high vacuum level in the preparation chamber (7). The extra vacuum chamber (6) was added to further decrease the vacuum level during deposition, typical vacuum ranges are indicated in millibars. Two type of pumps are employed, i.e., primary and turbo pumps and the chamber separations are skimmer cones for the first two chambers and inlets for the others.

**Figure 2:**
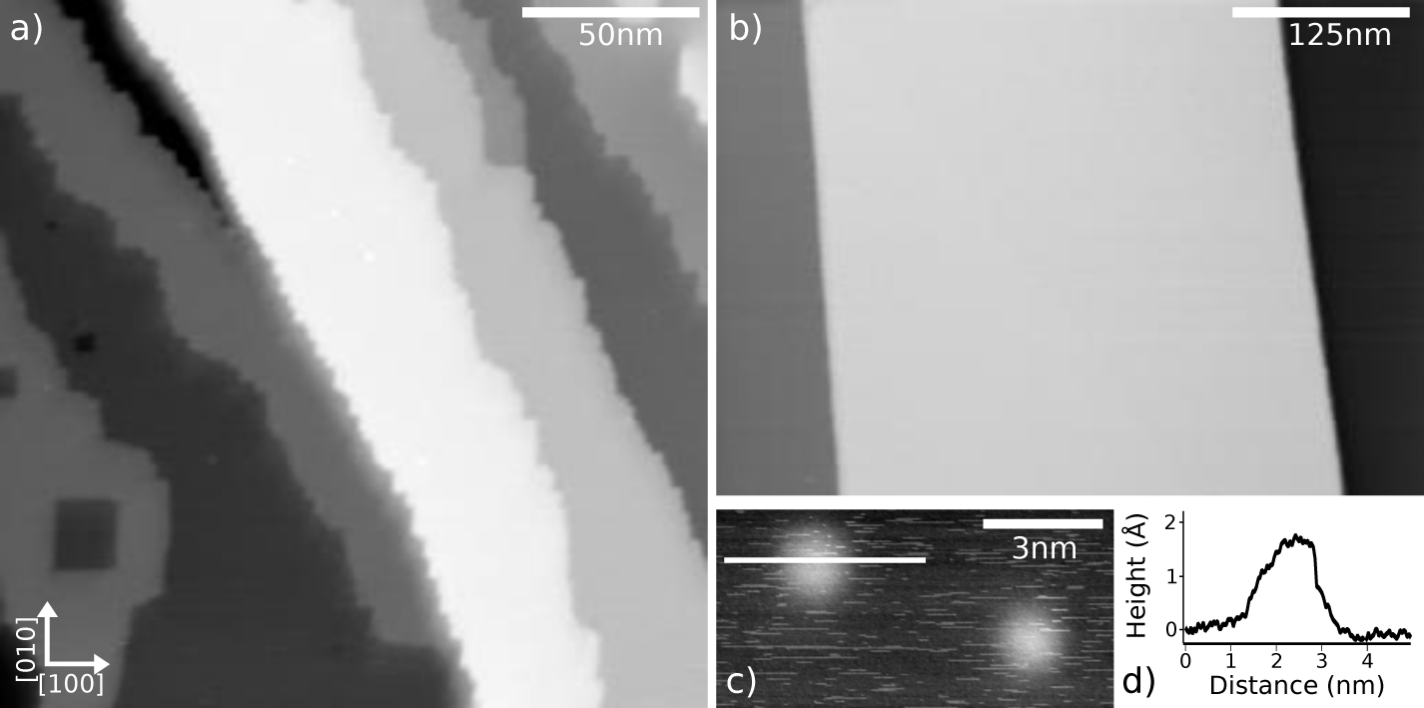
a) Topography image of the KBr(001) surface after the application of UHV-ESI with a mixture of toluene/isopropanol (2:1) over the course of 30 min. b) Topography image of clean KBr(001) surface obtained after UHV cleavage and following annealing at 400 K for 1h. c) Topography image of two protrusions with a profile (c) along the line. Parameters: a) *A* = 5 nm, Δ*f* = −10 Hz, b) *A* = 2 nm, Δ*f* = −60 Hz, c) *A* = 2 nm, Δ*f* = −25 Hz.

To confirm that residual solvent molecules introduced with the UHV-ESI process will not interfere with adsorbed molecules on the surface, we performed a deposition of the solvent solution only, i.e., toluene/isopropanol in the ratio 2:1 on a clean KBr(001) surface. Figure 2a shows a topography image acquired by nc-AFM at room temperature on a KBr(001) surface after a total time of 30 min of UHV-ESI deposition of the pure solvent. This is a long exposure time compared to the generally applied molecules deposition time of 1–5 min. As can be seen, terraces remain large and flat, and step-edges remain clean from adsorbates. However, some pits are observed on the terraces and step-edges present lots of kinks. Such pits are known from the bombardment of ionic surfaces by electrons or UV light and are attributed to a reorganization of color centers towards the surface [34].

The surface should be compared to the clean, UHV prepared, KBr(001) surface as presented in Figure 2b. Here, large terraces and step-edges aligned along the non-polar directions, e.g., [010] or [100] can be observed. Some protrusions can also be found on the surface, however their number remains small. In Figure 2b, a zoom on two of these protrusions is presented with a profile in Figure 2c. The protrusions all appear with similar shape, i.e., circular with a diameter of 2 nm maximum. Since the solvents we used were high purity solvents, excluding any pollution, these protrusions can be attributed to solvent molecules or clusters. Another indication of the presence of the solvent are the spikes observed on this image. Most probably solvent molecules are still on the surface but are diffusing to fast for nc-AFM imaging.

### Large coverage UHV-ESI of triply fused diporphyrins

**Figure 3:**
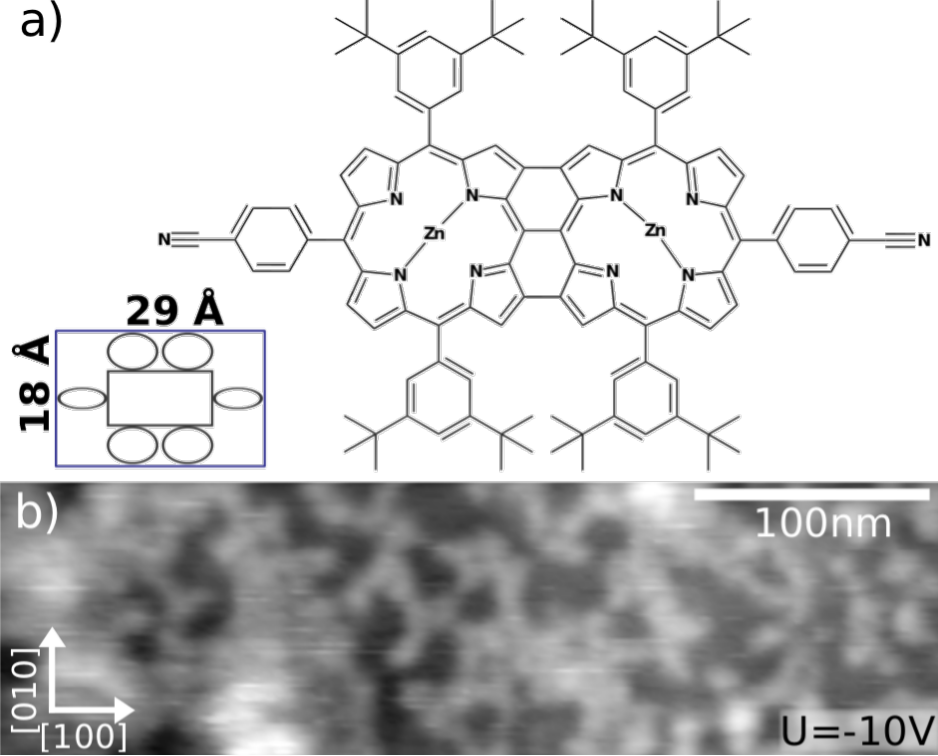
a) Chemical structure of the used triply fused diporphyrin molecule derivative prepared according to the synthetic protocols by D. Bonifazi et al. [39]. The inset shows a molecular scheme and the dimensions. b) Topography image after the UHV-ESI deposition of the porphyrins on KBr(001) for 5 min. Scan parameter: *A* = 2 nm, Δ*f* = −25 Hz, *U* = −10 V.

The complex porphyrin-based molecules under study are schematically shown in Figure 3a. It is a triply fused diporphyrin molecule including two 3-cyanophenyl groups and Zn metal cores. Similar molecules have already been thermally deposited on surfaces in UHV and are known to form self-assemblies on metallic substrates [35–36]. These molecules are rather complex compared to others, however the deposit by UHV-ESI can still be compared with the thermally deposited ones. Furthermore, the 3-cyanophenyl groups are known to enhance the binding to ionic substrates by electrostatic interaction [37–38]. For the UHV-ESI deposition, molecules were diluted at 1 μL/mL in a mixture of toluene and isopropanol with a 2:1 ratio. Deposition was performed for 5 min at a constant rate of injected solution of 5 μL/min, a maximum applied high voltage of 1.5 kV, and a pressure in the vacuum chamber below 2 × 10^−7^ mbar.

A topographic image acquired by nc-AFM after UHV-ESI deposition of the diporphyrin on the clean KBr(001) surface is displayed in Figure 3b. Large molecular features are homogeneously distributed all over the surface and appear up to 3 nm high. However, no clear organization of the molecules was observed. Large electrostatic forces have been observed and measured via frequency shift versus voltage curves d*f*(*V*) [40] after the deposition process. These could not be compensated during measurements by applying a bias voltage of up to ±10 V, which is the limit of the AFM system. These large electrostatic forces induced difficult scan conditions and we were not able to study the organization of the molecules in more detail. Indeed, surfaces charges of bulk insulator samples have already been studied in detail [41–42]. Due to cleavage many charges can be created resulting in large electrostatic forces. Sample preparation with soft annealing is well-established and leads to only a few remaining isolated charges [42]. Since the sample was prepared with such procedure before UHV-ESI deposition, we thus attribute this charging to the deposition process. During the deposition of only the solvent, we have not observed charging of the surface (Figure 2), whereas the presence of molecules in the solution always induces the surface charging for high coverage. Since the deposition time (minutes) is short compared to the charge compensation time (days), a charged surface is obtained. In our setup, a positive bias is applied to the solution containing the diporphyrin molecules. Therefore, droplets produced during ESI as well as the molecules reaching the surface are positively charged. This positive charging of the surface is in agreement with the estimated large negative bias voltages needed to compensate the surface potential of approximately −30 V.

A major disadvantage of UHV-ESI for insulating surfaces is thus the surface charging of the crystals, increasing with the deposition time and resulting in difficult scan conditions. High electrostatic forces in presence are also impacting the self-assembly of the molecules resulting in the molecular domains observed. Furthermore, above a certain amount, surface charges act as electrostatic barriers and prevent the landing of further molecules and high coverages are difficult to obtain.

### Triply fused diporphyrins UHV-ESI after annealing

**Figure 4:**
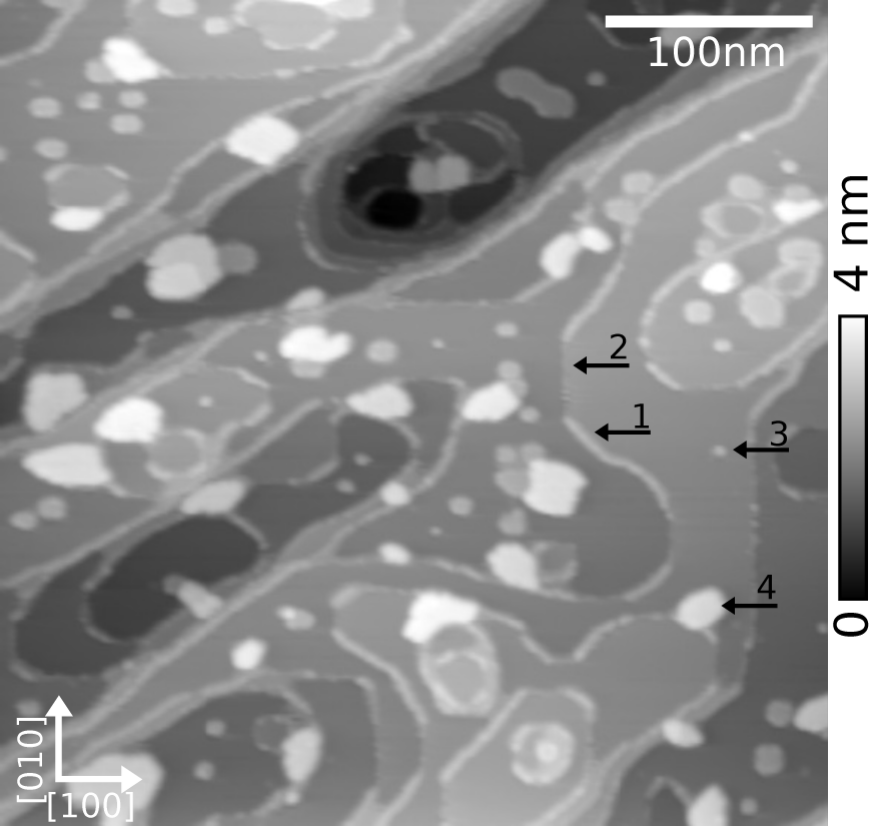
Topography image (400 × 400 nm^2^) of diporphyrins on KBr(001) after 1 h of annealing at 350 K. Different areas: covered (1) and non-covered (2) step edges, small aggregates (3), and molecular islands (4). Parameters: *A* = 4 nm, Δ*f* = −10 Hz.

Annealing of the sample was performed at 350 K for 1 h to remove the surface charges. Electrostatic force compensation was then reduced to −1.1 V, which is typical for KBr surfaces [43]. The topography image shown in Figure 4 reveals the surface modification due to the annealing in presence of the molecules. Indeed, pits, hills and a circular shape for the step-edges are observed instead of straight step-edges that are normally observed. Molecules are adsorbed at KBr step-edges (arrow 1) and also form small aggregates (arrow 3) at terraces or even larger islands (arrow 4). Their presence at step-edges is visible in Figure 4 as bright lines. Exceptions are step-edges oriented along the non-polar [010] and [100] directions (arrow 2), which are standard directions for KBr(001). The rounding of the normally straight step-edges is induced by the surface annealing in presence of molecules. Such phenomena have already been observed and described for truxene molecules containing similar cyanophenyl functional groups [31]. The circular shapes are created during deposition and annealing and allow the adsorbed molecules to be stabilized by reaching an overall energetic minimum. A result of this is the creation and stabilization of KBr pits and islands, which are not present on the untreated surface. Molecular islands can therefore be observed in theses pits [30] or on the KBr islands and present distinct height differences.

**Figure 5:**
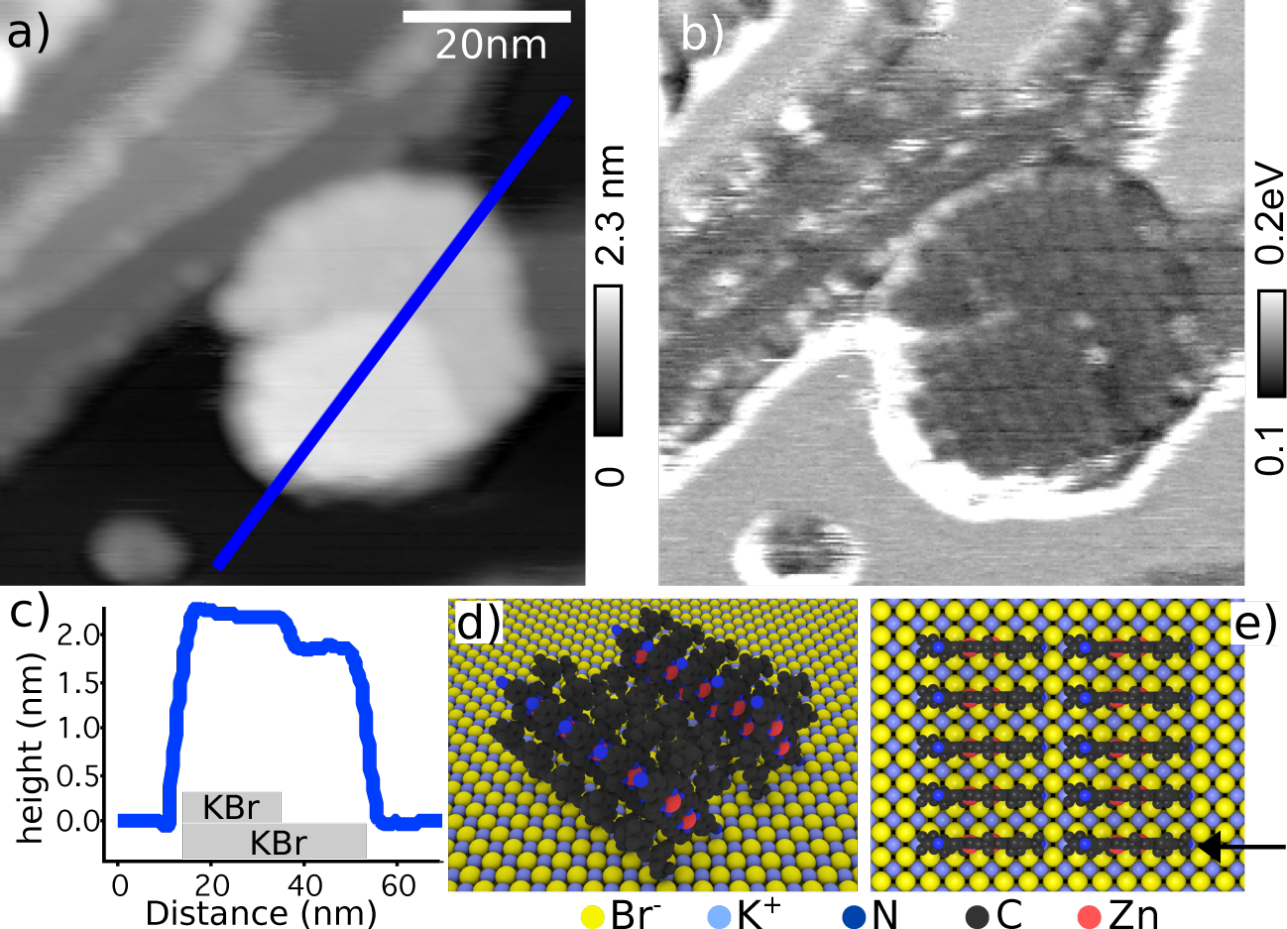
a) Topography image of a diporphyrin island on KBr(001). b) Corresponding dissipation image. c) Island profile, blue line in a). Scheme of the molecular arrangement in side view d) and in top view e). Black arrow point toward CN group anchored to K^+^ ions. Parameters: *A* = 5 nm, Δ*f* = −8 Hz.

A more detailed topography image of such an island is presented in Figure 5a with the corresponding dissipation image (Figure 5b). Using the dissipation image, the identification of the areas with and without molecules is facilitated due to two different contrasts (bright or dark). This phenomenon was already related [24]. The KBr terraces present a brighter contrast and molecules are observed in form of islands, at step-edges, in small aggregates and trapped in regions where successive step-edges are close. The profile presented in Figure 5c is acquired on the topography image and helps to understand the shape of the island. It shows two different heights, of 2.3 and 2.0 nm, compared to the KBr terrace. The difference corresponds to the height of a single KBr step meaning that the self-assembly partly sits on the KBr terrace and an island.

A better understanding of the molecular self-assembly is obtained by the dissipation image where single molecular rows are visible. A columnar organization of the rows with a length of 20–50 nm, and spaced by roughly 3 nm, compose the islands. This is in good agreement with the molecular size of 2.8 nm (Figure 5d,e) in a tilted position. Inside one molecular island and also for different islands, several line orientations can be observed (not shown here). Despite different tries and the intra islands resolution in the dissipation image, we were not able to achieve similar resolution in the topography images to know the exact commensurability between substrate and molecules. Few reasons can be mentioned related to a reduced imaging stability of this system. The molecules present a cyanophenyl group facing towards the tip and carrying a dipole moment which influences the electrostatic field distribution and with this also the imaging stability. Furthermore, this functional group is known to be quite flexible and will therefore, especially at room temperature conditions, prevent high-resolution imaging.

A schematic model of an island is proposed in Figure 5d and Figure 5e showing a possible molecular arrangement. In this configuration, columnar stabilization can be explained by a dominant molecule–molecule interaction by π–π-stacking [29,37,44–45]. Due to the symmetric arrangement of the two 3-cyanophenyl groups the electrostatic binding to the surface is only small and the molecular wires might easily flip or rearrange explaining the low stability in the AFM measurements and the different behaviour compared to smaller molecules [44–45]. The smaller features also visible at the surface can be attributed to molecular aggregates, but due to the high mobility at room temperature they were not investigated in more details.

### Low coverage of UHV-ESI porphyrins

To enable the analysis of single molecules, annealing of the sample should be avoided to restrict diffusion processes. Different parameters that could lead to a reduced amount of deposited molecules and charges can be tuned, like reducing deposition time or molecule concentration in the solution. Furthermore, deposition of solvent molecules should be reduced to a minimum which was achieved by the implementation of an additional pumping chamber to the spray setup (Figure 1), which helps to reduce the base pressure at the sample during spray deposition by an order of magnitude.

**Figure 6:**
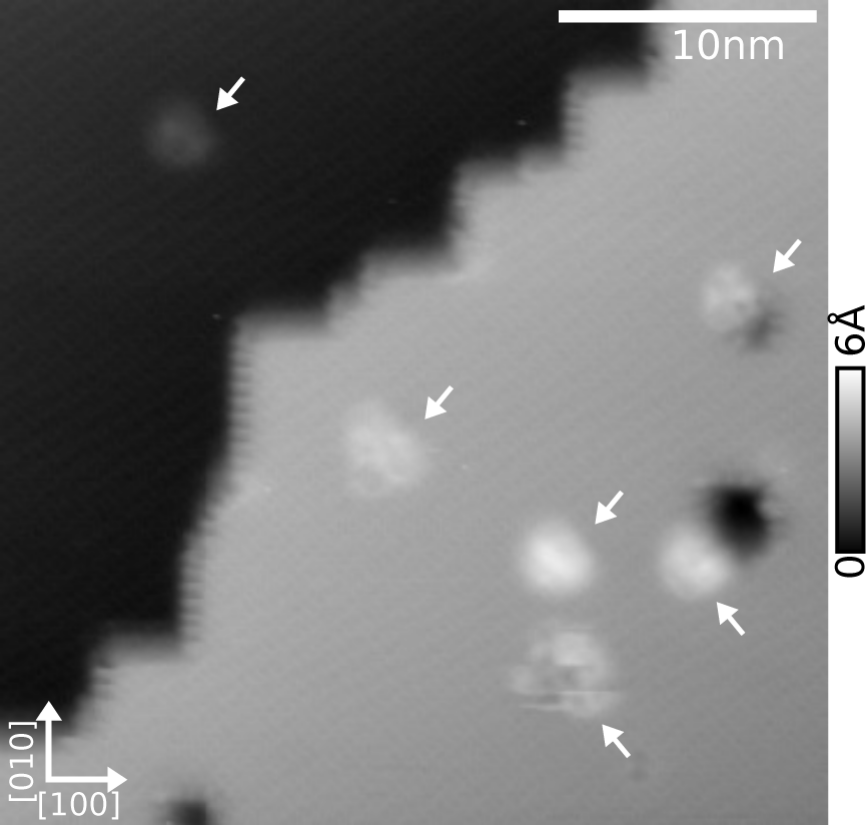
Topography image (40 × 40 nm^2^) of the KBr(001) surface with a low coverage of diporphyrin molecules deposited by UHV-ESI for 2 min. Parameters: *f*_2nd_ = 1.02 MHz, *A*_2nd_ = 400 pm, Δ*f*_2nd_ = −57 Hz.

Figure 6 presents the surface obtained after diporphyrin deposition at low coverage by UHV-ESI. Topography images is acquired at the 2nd flexural mode which allows one to enhance the sensitivity to short range forces by using small oscillation amplitude (400 pm) [46]. The topography shows two terraces separated by a kinked step-edge. Pits similar to what can be obtained after electron irradiation of the surface are visible [47]. Sample charging was not observed and bias remained in the ±1 V range. Small objects with sizes compatible with single molecules decorate both terraces. Due to the shorter deposition time, 2 min compared to 30 min in Figure 2, solvent molecules should only appear as traces on the surface and are unlikely to be observed here. However, two different contrasts have been observed, a stable and a slightly less stable one as can be seen in the distorted molecule at the bottom part of the image. Some of the single molecules are attached to the pits corners.

**Figure 7:**
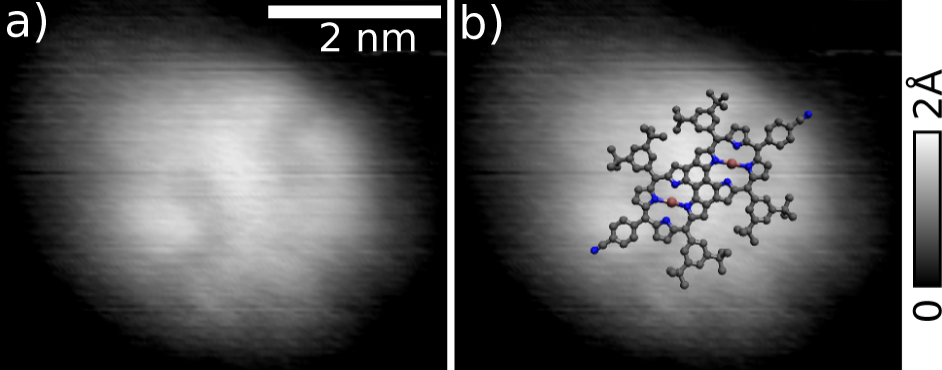
a) High resolution topography image of a single diporphyrin on KBr(001) at room temperature. b) Same image with added molecular model. Parameters: *f*_2nd_ = 1.02 MHz, *A*_2nd_ = 400 pm, Δ*f*_2nd_ = −70 Hz.

A high resolution image of a stable molecule presenting even the internal structure is shown in Figure 7. The image was acquired on the same sample but at another area. The simple superposition of the molecule drawing, is used to show that the observed object size fits with a single molecule. The molecule lay flat at the surface and binds through the 3-cyanophenyl groups to the KBr(001) substrate [29,31] indicated by the two symmetric protrusions. The two elongated features are attributable to the four 3,5-di(*tert*-butyl)phenyl moieties of the diporphyrin having a slightly higher topographic signature.

## Conclusion

The deposition of large functionalized molecules on surfaces with low contamination is important for fundamental studies. We show that UHV-ESI deposition, where molecules are contained in solution, fulfill theses conditions on insulating surfaces. This leads to the possibility to access molecular electronic properties at the single molecule level with scanning probe microscopy. We first demonstrated that solvent deposition from ESI has a weak influence on the KBr(001) surface. Then a complex molecule based on a triply fused diporphyrin was successfully deposited at various coverages on the KBr(001) surface. For a coverage of more than a few isolated molecules per 10 × 10 nm^2^ we have observed charging of the sample due to ion deposition. This charging can be easily overcome by annealing, leading to the formation of large molecular islands. We characterized theses islands showing molecular assemblies stabilized by π–π stacking organization and anchoring through the cyano group of the molecules and K^+^ ions of the surface. To achieve single molecule deposition, we added a pumping chamber in the setup that lowered the number of molecules reaching the surface. Due to the small amount of species on the surface we lowered the charging effect. In this way, we demonstrated the deposition of single molecules, down to few units per 100 × 100 nm^2^. For some adsorption geometries, where the molecules are laying flat on the surface, we obtained intra-molecular resolution at room temperature.

## Experimental

All experiments were performed under UHV conditions (*p* < 10^−10^ bar) with our home-built non-contact atomic force microscope (nc-AFM), operating at room temperature (RT) [48]. Bulk insulator KBr(001) crystals surfaces (from MaTeck GmbH) were prepared in situ by cleavage followed with an annealing at 400 K for 1 h to remove residual charges. PPP-NCL cantilevers (Nanosensor) with typical resonance frequency of *f*_1st_ ≈ 170 kHz and first harmonic of *f*_2nd_ ≈ 1 MHz were used. Sensor preparation consists of annealing for 1 h at 400 K and tip sputtering for 90 s at 680 eV at an Ar^+^ pressure of *p* = 3 × 10^−6^ bar.

The ESI setup (Figure 1) is connected to the UHV preparation chamber of the system. It is a commercial system from MolecularSpray [14,33]. After the spray is formed in air, highly charged droplets [49–51] enter by a capillary into the differential pumping system composed of the three chambers separated by leak orifices. During this differential pumping system, initial droplets undergo successive droplet solvent pumping and coulomb fission leading to ionized molecules only. The spray quality in air was controlled during deposition with a camera. The base pressure in the sample chamber was in the low 10^−10^ mbar range, and increase to low *p* ≈ 10^−7^ mbar during spray deposition. Typical parameters for UHV-ESI are 1.3–2.0 kV, sometimes adjusted to maintain spray quality during deposition. The deposition time was tuned to obtain different coverages between 1–30 min at controlled flux, by the use of a syringe pump, of 2–10 μL/min. The molecules were diluted in a solution of toluene/isopropanol made from high purity solvents (Sigma-Aldrich) with a ratio of 2:1. Molecule concentration in solution was 1 μg/mL.

Molecules, see Figure 3, are based on a triply fused double porphyrin and include two metallic core atoms (zinc) and two cyanophenyl groups. More information on the synthesis of the molecules can be found in [35–36,39]

## References

[1] O’Regan B, Grätzel M (1991). Nature.

[2] Joachim C, Gimzewski J K, Aviram A (2000). Nature.

[3] Pang C L, Ishibashi T-a, Onishi H (2005). Jpn J Appl Phys.

[4] Tanaka H, Kawai T (1997). J Vac Sci Technol, B.

[5] Zambelli T, Boutayeb Y, Gayral F, Lagoute J, Girdhar N K, Gourdon A, Gauthier S, Blanco M-J, Chambron J-C, Heitz V (2004). Int J Nanosci.

[6] Fenn J B, Mann M, Meng C K, Wong S F, Whitehouse C M (1989). Science.

[7] Rauschenbach S, Stadler F L, Lunedei E, Malinowski N, Koltsov S, Costantini G, Kern K (2006). Small.

[8] Hamann C, Woltmann R, Hong I-P, Hauptmann N, Karan S, Berndt R (2011). Rev Sci Instrum.

[9] Kley C S, Dette C, Rinke G, Patrick C E, Čechal J, Jung S J, Baur M, Dürr M, Rauschenbach S, Giustino F (2014). Nano Lett.

[10] Hauptmann N, Scheil K, Gopakumar T G, Otte F L, Schütt C, Herges R, Berndt R (2013). J Am Chem Soc.

[11] Rauschenbach S, Vogelgesang R, Malinowski N, Gerlach J W, Benyoucef M, Costantini G, Deng Z, Thontasen N, Kern K (2009). ACS Nano.

[12] Hauptmann N, Hamann C, Tang H, Berndt R (2013). J Phys Chem C.

[13] Bodin A, Laloo R, Abeilhou P, Guiraud L, Gauthier S, Martrou D (2013). Rev Sci Instrum.

[14] Satterley C J, Perdigão L M A, Saywell A, Magnano G, Rienzo A, Mayor L C, Dhanak V R, Beton P H, O’Shea J N (2007). Nanotechnology.

[15] Saywell A, Magnano G, Satterley C J, Perdigão L M A, Champness N R, Beton P H, O’Shea J N (2008). J Phys Chem C.

[16] Saywell A, Sprafke J K, Esdaile L J, Britton A J, Rienzo A, Anderson H L, O’Shea J N, Beton P H (2010). Angew Chem, Int Ed.

[17] Rienzo A, Mayor L C, Magnano G, Satterley C J, Ataman E, Schnadt J, Schulte K, O’Shea J N (2010). J Chem Phys.

[18] Handrup K, Richards V J, Weston M, Champness N R, O’Shea J N (2013). J Chem Phys.

[19] Svatek S A, Perdigão L M A, Stannard A, Wieland M B, Kondratuk D V, Anderson H L, O’Shea J N, Beton P H (2013). Nano Lett.

[20] Wieland M B, Perdigão L M A, Kondratuk D V, O’Shea J N, Anderson H L, Beton P H (2014). Chem Commun.

[21] Pfeiffer O, Gnecco E, Zimmerli L, Maier S, Meyer E, Nony L, Bennewitz R, Diederich F, Fang H, Bonifazi D (2005). J Phys: Conf Ser.

[22] Kunstmann T, Schlarb A, Fendrich M, Wagner T, Möller R, Hoffmann R (2005). Phys Rev B.

[23] Mativetsky J M, Burke S A, Fostner S, Grütter P (2007). Small.

[24] Hinaut A, Lekhal K, Aivazian G, Bataillé S, Gourdon A, Martrou D, Gauthier S (2011). J Phys Chem C.

[25] Pawlak R, Nony L, Bocquet F, Oison V, Sassi M, Debierre J-M, Loppacher C, Porte L (2010). J Phys Chem C.

[26] Neff J L, Götzen J, Li E, Marz M, Hoffmann-Vogel R (2012). Beilstein J Nanotechnol.

[27] Kittelmann M, Rahe P, Gourdon A, Kühnle A (2012). ACS Nano.

[28] Schütte J, Bechstein R, Rohlfing M, Reichling M, Kühnle A (2009). Phys Rev B.

[29] Hinaut A, Pujol A, Chaumeton F, Martrou D, Gourdon A, Gauthier S (2012). Beilstein J Nanotechnol.

[30] Nony L, Gnecco E, Baratoff A, Alkauskas A, Bennewitz R, Pfeiffer O, Maier S, Wetzel A, Meyer E, Gerber C (2004). Nano Lett.

[31] Such B, Trevethan T, Glatzel T, Kawai S, Zimmerli L, Meyer E, Shluger A L, Amijs C H M, de Mendoza P, Echavarren A M (2010). ACS Nano.

[32] Trevethan T, Such B, Glatzel T, Kawai S, Shluger A L, Meyer E, de Mendoza P, Echavarren A M (2011). Small.

[33] (2015). Molecularspray Ltd..

[34] Such B, Czuba P, Piatkowski P, Szymonski M (2000). Surf Sci.

[35] Bonifazi D, Spillmann H, Kiebele A, de Wild M, Seiler P, Cheng F, Güntherodt H-J, Jung T, Diederich F (2004). Angew Chem, Int Ed.

[36] Bonifazi D, Kiebele A, Stöhr M, Cheng F, Jung T, Diederich F, Spillmann H (2007). Adv Funct Mater.

[37] Maier S, Fendt L-A, Zimmerli L, Glatzel T, Pfeiffer O, Diederich F, Meyer E (2008). Small.

[38] Zimmerli L, Maier S, Glatzel T, Gnecco E, Pfeiffer O, Diederich F, Fendt L, Meyer E (2007). J Phys: Conf Ser.

[39] Bonifazi D, Scholl M, Song F, Echegoyen L, Accorsi G, Armaroli N, Diederich F (2003). Angew Chem.

[40] Guggisberg M, Bammerlin M, Loppacher C, Pfeiffer O, Abdurixit A, Barwich V, Bennewitz R, Baratoff A, Meyer E, Güntherodt H-J (2000). Phys Rev B.

[41] Barth C, Henry C R (2007). Phys Rev Lett.

[42] Barth C, Henry C R (2006). Nanotechnology.

[43] Bocquet F, Nony L, Loppacher C, Glatzel T (2008). Phys Rev B.

[44] Glatzel T, Zimmerli L, Koch S, Kawai S, Meyer E (2009). Appl Phys Lett.

[45] Glatzel T, Zimmerli L, Kawai S, Meyer E, Fendt L-A, Diederich F (2011). Beilstein J Nanotechnol.

[46] Kawai S, Kitamura S-i, Kobayashi D, Meguro S, Kawakatsu H (2005). Appl Phys Lett.

[47] Kolodziej J J, Such B, Czuba P, Krok F, Piatkowski P, Struski P, Szymonski M, Bennewitz R, Schär S, Meyer E (2001). Surf Sci.

[48] Howald L, Meyer E, Lüthi R, Haefke H, Overney R, Rudin H, Güntherodt H-J (1993). Appl Phys Lett.

[49] Kebarle P, Tang L (1993). Anal Chem.

[50] Fenn J B (1993). J Am Soc Mass Spectrom.

[51] Gaskell S J (1997). J Mass Spectrom.

[52] Horcas I, Fernández R, Gómez-Rodríguez J M, Colchero J, Gómez-Herrero J, Baro A M (2007). Rev Sci Instrum.

